# The Restoration of Hahnemann’s Tomb

**Published:** 1899-12

**Authors:** Bushrod W. James


### THE RESTORATION OF HAHNEMANN'S TOMB.

The total amount of contributions received by Dr. Cartier, for the restoration of Hahnemann's tomb, in Paris, is 17,424.40 francs.

The design adopted was that of Lardot, and according to Dr. Cartier's description in the October number of the *Revue Homœopathique*, is as follows: "The monument is composed of a central piece and two lower sides. In the centre is a pedestal, ornamented with carvings and bronze garlands, which supports Hahnemann's bust; back of the pedestal a large stela (arch) surmounted by carved emblems, and of 3.80 m. in height; on the body of the stela is Hahnemann's epitaph; at the foot of the pedestal is read 'International Subscriptions.' "

On the sides are engraved, on the left, the works of Hahnemann; on the right, his sentiments.

"The side of the base on which is engraved the works and sentiments, is further ornamented with palm leaves, consols, and placques in reliefs, for engraving letters.

"In front of the monument are double perpend stones, molded to hold a railing in antique green bronze, Greek style.

"The monument will be of Scotch red granite, from Peterhead of imperishable polish, except the sub-base, which will be of Normandy granite, probably Vire.

"In the agreement with the house of Lardot, the monument must be finished for the International Congress of 1900, which will be held at the Exposition from the 18th to the 21st of July.

"Subscriptions will be received until December 31st, 1899, as certain parts of the tomb can be much more richly ornamented."

Those who desire to contribute have therefore the privilege of sending in amounts up to the first of January, 1900.

The fund in hand covers the contract already made for the restoration, but some further ornamentation should be added to the monument and any additional subscriptions will be used for this purpose.

The French society is pleased that the American physicians have taken so much interest in this restoration of Hahnemann's tomb, especially in view of the grand monument which is contemplated being placed in Washington.

As the American member of the commission, I desire to thank the physicians of our country for the deep interest which they have manifested in this measure.

The monument will be dedicated during the International Congress to be held in Paris in July, 1900.

BUSHROD W. JAMES.

